# Hydroxychloroquine-Induced Renal Phospholipidosis

**DOI:** 10.7759/cureus.96790

**Published:** 2025-11-13

**Authors:** Khazeema Hafeez, Stephan Baker, Sandeep K Shori, Sampada Acharya, Shovendra Gautam

**Affiliations:** 1 Internal Medicine, Baylor Scott & White All Saints Medical Center, Fort Worth, USA; 2 Pathology, Baylor Scott & White All Saints Medical Center, Fort Worth, USA; 3 Nephrology, Baylor Scott & White All Saints Medical Center, Fort Worth, USA; 4 Rheumatology, Baylor Scott & White All Saints Medical Center, Fort Worth, USA

**Keywords:** hydroxychloroquine, lamellar bodies, nephrotic range proteinuria, phospholipidosis, sjogren's

## Abstract

Renal phospholipidosis is a rare condition in which excessive phospholipids accumulate in the lysosomes of the renal cells, leading to renal dysfunction. Historically, phospholipidosis has been described in the context of inherited disorders of lysosomal phospholipid metabolism such as Fabry disease. It is now known that many medications can cause excessive phospholipid accumulation, leading to cellular dysfunction. We present a case of hydroxychloroquine-induced renal phospholipidosis in a 79-year-old female patient with Sjogren’s disease who had been on hydroxychloroquine for several years and developed nephrotic range proteinuria. She underwent a renal biopsy that showed myeloid (lamellar) bodies within podocytes, a morphological sign of phospholipidosis. Immunofluorescence staining for other potential causes was negative, making it a case of drug-induced phospholipidosis. This patient also had underlying chronic kidney disease stage II. This case raises a question of whether an underlying impaired renal function contributes to the risk of developing drug-induced phospholipidosis, as well as the importance of timely and accurate diagnosis to prevent further harm and to increase the chances of renal recovery.

## Introduction

Renal phospholipidosis is a rare condition characterized by excessive accumulation of phospholipids in the lysosomes of the kidney, particularly in the podocytes. This excessive accumulation causes kidney damage, leading to proteinuria and renal dysfunction. Phospholipidosis has primarily been described in lysosomal storage disorders such as Fabry disease, but we now know that certain medications such as amiodarone, fluoxetine, gentamycin, hydroxychloroquine, and perhexiline can lead to an acquired form of phospholipidosis [1,2]. In drug-induced phospholipidosis (DIPL), the offending medication alters lipid metabolism by an unclear mechanism and leads to excessive intracellular accumulation of phospholipids in multiple organs of the body, including the kidney, liver, lung, brain, cornea, and other organs [3].

Drug-induced phospholipidosis was first reported in 1948 by Nelson and Fitzhugh in rats treated with chloroquine [4]. Over the last 60 years, more than 50 medications have been associated with phospholipidosis [4,5]. The exact mechanism of drug-induced phospholipidosis is unclear, but there are a few hypotheses. The first hypothesis suggests that the offending medication binds directly to phospholipids, leading to a drug-lipid complex that accumulates to form phospholipid lamellar bodies. A second hypothesis suggests that the offending medication inhibits phospholipase, an enzyme that breaks down phospholipids, resulting in phospholipid accumulation [2,6]. Regardless of the mechanism, organs affected by phospholipidosis exhibit inflammatory reactions and histopathological changes, such as macrophagic infiltration or fibrosis [2,7]. We report a case of drug-induced renal phospholipidosis in a patient who developed nephrotic range proteinuria after long-term use of hydroxychloroquine.

## Case presentation

A 79-year-old female patient with past medical history of chronic kidney disease Stage II and Sjogren’s disease, diagnosed five years ago when she initially presented with joint pain, dry eyes, dry mouth, and persistent cough, and had serological evidence of anti-nuclear antibodies (ANA), anti-SSA(Ro), and anti-SSB(La). She had been on a stable dose of hydroxychloroquine 400 mg daily for the past five years, but recently the dose was decreased to 200 mg daily to prevent long-term adverse effects in the future. However, two months after reducing the dose patient developed an erythematous rash with some blisters mainly over the neck, which prompted the hydroxychloroquine dose to be increased back to 400 mg daily. Her renal function, including estimated glomerular filtration rate (eGFR) and baseline creatinine, has been stable over the past five years (Table 1).

**Table 1:** Renal function panel and other pertinent labs BUN: blood urea nitrogen; eGFR: estimated glomerular filtration rate

Lab	Patient value	Reference value
Sodium	140 mmol/L	135-146 mmol/L
Potassium	4 mmol/L	3.5-5.3 mmol/L
Chloride	100 mmol/L	98-110 mmol/L
Bicarbonate	31 mmol/L	20-32 mmol/L
Glucose	108 mg/dL	65-99 mg/dL
BUN	18 mg/dL	7-25 mg/dL
Creatinine	0.79 mg/dL	0.6-1.00 mg/dL
eGFR	76 mL/min/1.73m2	> OR = 60 mL/min/1.73m2
Albumin	4.7 g/dL	3.6-5.1 g/dL
Hemoglobin A1C	5.6 %	< 5.7%
Uric acid	5 mg/dL	2.5-7.0 mg/dL

However, her urine protein had slowly increased over the past year and was found to have nephrotic range proteinuria on her most recent routine urine chemistry (Tables 2, 3).

**Table 2:** Urine protein/creatinine ratio

Lab	Patient value	Reference value
Urine protein (random)	536 mg/dL	5-24 mg/dL
Urine creatinine (random)	114 mg/dL	20-275 mg/dL
Urine protein/creatinine ratio (random)	4,702 mg/g creatinine	24-184 mg/g creatinine

**Table 3:** Urinalysis

Lab	Patient value	Reference value
Color, Urine	Yellow	Yellow
Appearance U	Clear	Clear
Specific gravity	1.019	1.001-1.035
pH urine	5.5	5.0-8.0
Glucose	Negative	Negative
Ketones	Negative	Negative
Hemoglobin urine	Negative	Negative
Protein	3+	Negative

There was no serological evidence of increased disease activity of Sjogren’s (Table 4), as well as a low EULAR Sjögren's Syndrome Disease Activity Index (ESSDAI). 

**Table 4:** Serological and inflammatory markers SSA/Ro: Sjögren anti-SSA/Ro antibody; SSB/La; Sjögren anti-SSB/La antibody; RF: rheumatoid factor; CCP: cyclic citrullinated peptide antibody; ANA: antinuclear antibody; ESR: erythrocyte sediment rate; CRP: C-reactive protein

Lab	Patient value	Reference value
SSA/Ro	Negative	Negative
SSB/La	Negative	Negative
RF	10 IU/mL	0-13 IU/mL
CCP	Negative	Negative
CCP quantitative	< 0.5 U/mL	0.0 – 2.9 U/mL
ANA	Negative	Negative
ESR	21 mm/h	0-20 mm/h
CRP	< 0.3 mg/dL	0.0-0.4 mg/dL

**Figure 1:**
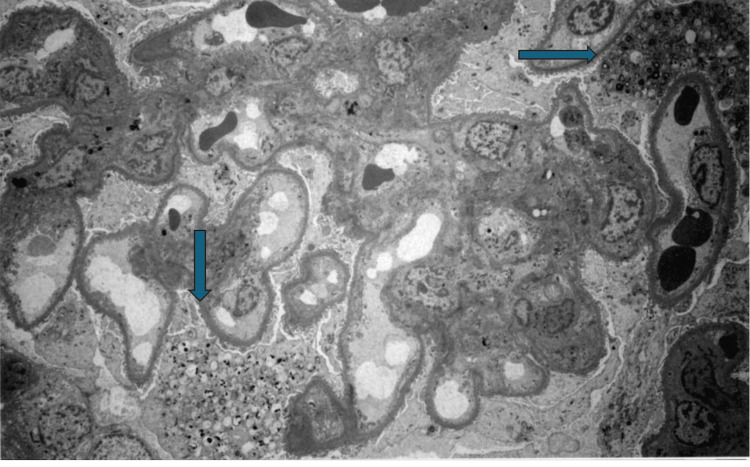
Electron micrograph demonstrating myeloid (lamellar) bodies within podocyte and parietal epithelial cell cytoplasm (1400x)

**Figure 2:**
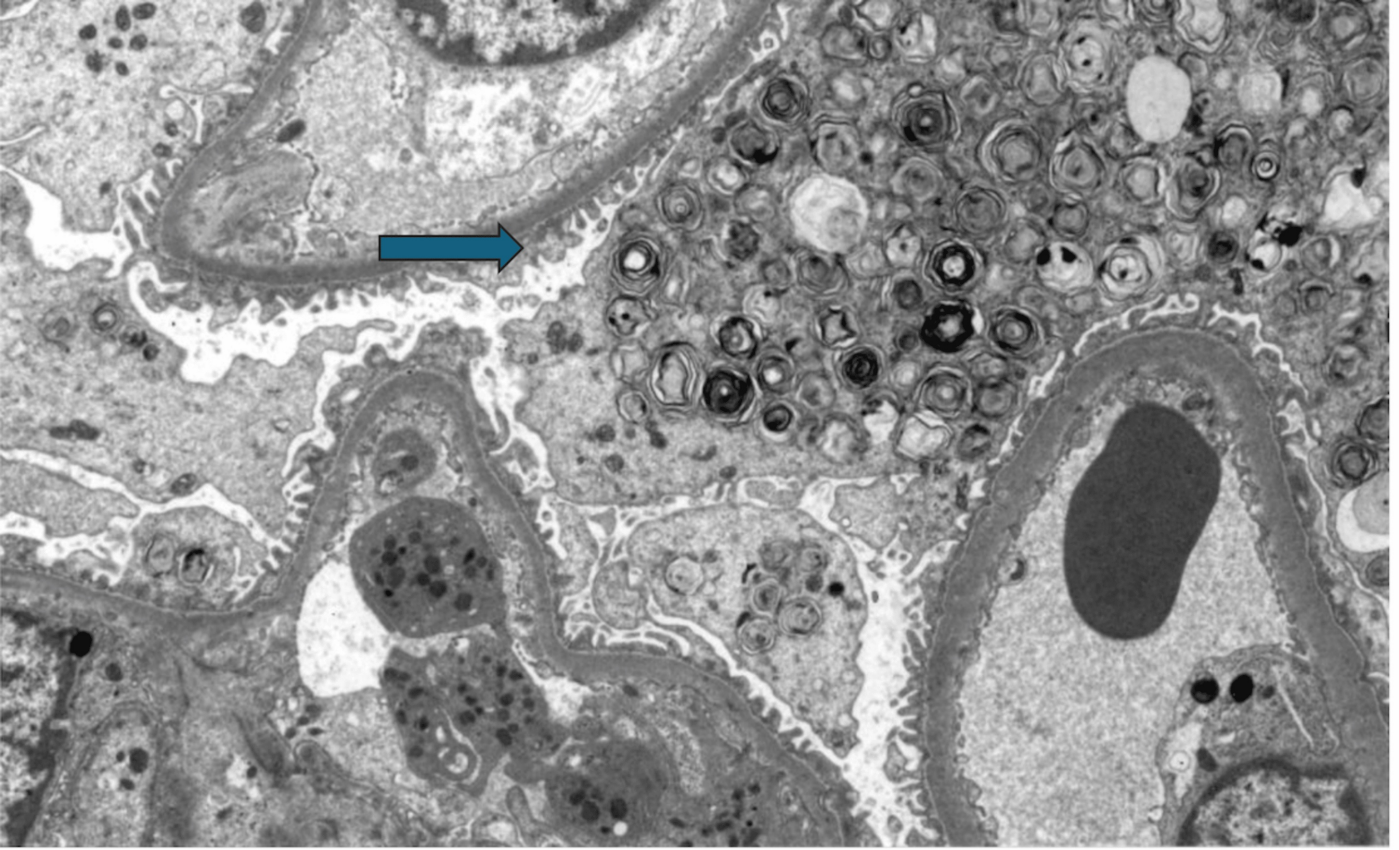
Electron micrograph with higher power view of myeloid (lamellar) bodies within podocyte and parietal epithelial cell cytoplasm (4800x)

Patient underwent renal biopsy that showed effacement of foot processes with prominent lamellar bodies in podocytes, a morphological sign of phospholipidosis (Figures 1, 2). Immunofluorescence staining for IgG, IgA, IgM, C3, C1q, kappa, lambda, fibrinogen, and albumin was all negative, making it more likely to be drug-induced phospholipidosis. The patient was diagnosed with renal phospholipidosis secondary to long-term hydroxychloroquine use. Hydroxychloroquine was discontinued, and the patient was transitioned to azathioprine. Renal function improved significantly after discontinuing the hydroxychloroquine. Repeat labs three months after stopping the offending agent showed a significant reduction in proteinuria and improvement in renal function, reiterating the importance of early and accurate diagnosis. Renal function improved significantly after discontinuation of hydroxychloroquine. Repeat labs three months after stopping the offending agent showed a significant reduction in proteinuria and improvement in renal function.

## Discussion

Case studies have demonstrated a strong link between chronic kidney disease (CKD) and lysosomal storage diseases, especially Fabry disease. A case series published in Annals of Medical and Health Sciences Research demonstrated Fabry disease to be a rare cause of end-stage renal disease [7]. There have also been numerous case studies that demonstrate how certain medications, including hydroxychloroquine, can mimic Fabry disease and cause renal dysfunction [2]. There have also been studies that suggest chronic kidney disease can lead to decreased renal excretion of these drugs, leading to increased drug accumulation and drug toxicity [8].

Hydroxychloroquine is frequently used to treat primary Sjogren syndrome and other rheumatological conditions. Various mechanisms of action have been hypothesized to explain the therapeutic and adverse effects of hydroxychloroquine. An important mode of action of the drug involves lysosomal activity and autophagy, which might perhaps also explain phospholipidosis, one of the adverse effects of the medication. Hydroxychloroquine accumulates in the lysosome and inhibits its function [9]. Lysosomes are acidic organelles that are not only responsible for breaking down waste material but are also involved in antigen processing and major histocompatibility complex (MHC) class II presentation. Hydroxychloroquine prevents lysosomal MHC class II-mediated autoantigen presentation, thereby modulating the immune system and preventing the activation of autoreactive CD4+ T cells, thus leading to anti-inflammatory effects [9].

Another important function of the lysosome is phospholipid metabolism, which was appreciated with the recognition of the inherited deficiencies of lysosomal storage diseases such as Niemann-Pick disease and Fabry disease [5]. In lysosomal storage diseases, an inherited defect in the enzyme causes intracellular accumulation of the enzyme substrate, leading to cellular damage. A similar mechanism is noted in drug-induced phospholipidosis that leads to drug-mediated cellular toxicity. The mechanism of drug-induced phospholipidosis is not well understood, but it is known that cationic amphiphilic drugs (CADs) such as hydroxychloroquine are lysosomotropics. They have a hydrophilic amine head group that can be protonated in the endolysosomal compartment and carry a positive charge, while the hydrophobic tail consisting of an aromatic or aliphatic ring structure can anchor in lipid bilayers of the cellular membrane. These amphiphiles then get trapped in the lysosome [3,10]. A low pH is optimal for lysosomal enzymes involved in hydrolysis. As these cationic amphiphilic drugs (CADs) accumulate in the lysosome, they increase the pH and inhibit lysosomal activity, thereby not only preventing MHC class II-mediated autoantigen presentation but also preventing phospholipid metabolism, leading to phospholipidosis [9]. As phospholipids build up in the cellular compartments, they trigger an inflammatory response and lead to tissue and organ damage. These inflammatory reactions and histopathological changes can be seen as macrophagic infiltration or fibrosis [2]. The affected cells appear foamy, and the cytoplasm is classically marked by the appearance of myeloid bodies. Histologically, this appearance is referred toas lamellar bodies, also called zebra bodies due to their striped morphology [5].

## Conclusions

This case raises a question of when to suspect drug-induced phospholipidosis, particularly in patients who do not exhibit any signs and symptoms of organ toxicity, such as our patient, who was incidentally found to have proteinuria on routine labs. This highlights the imperative for clinicians to carefully review medication history and perform a routine component of diagnostic workup to monitor potential toxicity, especially with long-term use of medications with a well-documented history of drug-induced phospholipidosis. This case also raises the question of whether an underlying impaired renal function contributes to the risk of developing drug-induced phospholipidosis. Finally, a comprehensive diagnostic approach should be taken when drug-induced phospholipidosis is suspected to rule out other potential causes and to achieve an accurate diagnosis so the offending medication can be stopped in a timely manner to increase chances of organ recovery. In our patient, renal function improved significantly after discontinuing the hydroxychloroquine. Repeat labs three months after stopping the offending agent showed a significant reduction in proteinuria and improvement in renal function, reiterating the importance of early and accurate diagnosis.

## References

[1] (2025). New Zealand data sheet - Plaquenil. https://www.medsafe.govt.nz/profs/datasheet/p/Plaqueniltab.pdf.

[2] Anderson N, Borlak J (2006). Drug-induced phospholipidosis. FEBS Lett.

[3] Breiden B, Sandhoff K (2019). Emerging mechanisms of drug-induced phospholipidosis. Biol Chem.

[4] Fitzhugh OG, Nelson AA, Holland OL (1948). The chronic oral toxicity of chloroquine. J Pharmacol Exp Ther.

[5] Shayman JA, Abe A (2013). Drug induced phospholipidosis: an acquired lysosomal storage disorder. Biochim Biophys Acta.

[6] Robison RL, Visscher GE, Roberts SA, Engstrom RG, Hartman HA, Ballard FH (1985). Generalized phospholipidosis induced by an amphiphilic cationic psychotropic drug. Toxicol Pathol.

[7] Wani MM, Khan I, Bhat RA, Ahmad M (2016). Fabry's disease: case series and review of literature. Ann Med Health Sci Res.

[8] Browning DJ (2014). Pharmacology of chloroquine and hydroxychloroquine. Hydroxychloroquine and Chloroquine Retinopathy.

[9] Dake MD, Madison JM, Montgomery CK, Shellito JE, Hinchcliffe WA, Winkler ML, Bainton DF (1985). Electron microscopic demonstration of lysosomal inclusion bodies in lung, liver, lymph nodes, and blood leukocytes of patients with amiodarone pulmonary toxicity. Am J Med.

[10] Schrezenmeier E, Dörner T (2020). Mechanisms of action of hydroxychloroquine and chloroquine: implications for rheumatology. Nat Rev Rheumatol.

